# Protein O-Glucosyltransferase 1 (POGLUT1) Promotes Mouse Gastrulation through Modification of the Apical Polarity Protein CRUMBS2

**DOI:** 10.1371/journal.pgen.1005551

**Published:** 2015-10-23

**Authors:** Nitya Ramkumar, Beth M. Harvey, Jeffrey D. Lee, Heather L. Alcorn, Nancy F. Silva-Gagliardi, C. Jane McGlade, Timothy H. Bestor, Jan Wijnholds, Robert S. Haltiwanger, Kathryn V. Anderson

**Affiliations:** 1 Developmental Biology Program, Sloan Kettering Institute, Memorial Sloan Kettering Cancer Center, New York, New York, United States of America; 2 Program in Biochemistry and Structural Biology, Cell and Developmental Biology, and Molecular Biology, Weill Cornell Graduate School of Medical Sciences, Cornell University, New York, New York, United States of America; 3 Department of Biochemistry and Cell Biology, Stony Brook University, Stony Brook, New York, United States of America; 4 The Hospital for Sick Children, Arthur and Sonia Labatt Brain Tumor Research Center and Department of Medical Biophysics, University of Toronto, Toronto, Ontario, Canada; 5 Department of Genetics and Development, College of Physicians and Surgeons of Columbia University, New York, New York, United States of America; 6 Department of Neuromedical Genetics, Netherlands Institute for Neuroscience, Amsterdam, The Netherlands; University of Virginia, UNITED STATES

## Abstract

Crumbs family proteins are apical transmembrane proteins with ancient roles in cell polarity. Mouse *Crumbs2* mutants arrest at midgestation with abnormal neural plate morphology and a deficit of mesoderm caused by defects in gastrulation. We identified an ENU-induced mutation, *wsnp*, that phenocopies the *Crumbs2* null phenotype. We show that *wsnp* is a null allele of *Protein O-glucosyltransferase 1* (*Poglut1*), which encodes an enzyme previously shown to add O-glucose to EGF repeats in the extracellular domain of *Drosophila* and mammalian Notch, but the role of POGLUT1 in mammalian gastrulation has not been investigated. As predicted, we find that POGLUT1 is essential for Notch signaling in the early mouse embryo. However, the loss of mouse POGLUT1 causes an earlier and more dramatic phenotype than does the loss of activity of the Notch pathway, indicating that POGLUT1 has additional biologically relevant substrates. Using mass spectrometry, we show that POGLUT1 modifies EGF repeats in the extracellular domain of full-length mouse CRUMBS2. CRUMBS2 that lacks the O-glucose modification fails to be enriched on the apical plasma membrane and instead accumulates in the endoplasmic reticulum. The data demonstrate that CRUMBS2 is the target of POGLUT1 for the gastrulation epithelial-to-mesenchymal transitions (EMT) and that all activity of CRUMBS2 depends on modification by POGLUT1. Mutations in human *POGLUT1* cause Dowling-Degos Disease, POGLUT1 is overexpressed in a variety of tumor cells, and mutations in the EGF repeats of human CRUMBS proteins are associated with human congenital nephrosis, retinitis pigmentosa and retinal degeneration, suggesting that O-glucosylation of CRUMBS proteins has broad roles in human health.

## Author Summary

Post-translational addition of sugar chains is essential for normal activity of many secreted and transmembrane proteins and dozens of human genetic diseases are associated with congenital disorders of glycosylation. Protein O-glucosyltransferase 1 (POGLUT1), which is essential for early mouse development, catalyzes the addition of O-glucose to extracellular EGF repeats of proteins, including NOTCH1. Here we show that mouse POGLUT1 modifies NOTCH1 *in vivo*; however, the essential role of POGLUT1 in gastrulation is due to POGLUT1-dependent glycosylation of EGF repeats in the apical polarity protein CRUMBS2. In contrast to findings in *Drosophila*, where modification of Crumbs by POGLUT1 is not required, mouse POGLUT1 is required for the activity of CRUMBS2: the unmodified protein fails to localize to the apical membrane and the gastrulation defects of *Poglut1* mutants are indistinguishable from those of *Crumbs2* mutants. Human mutations in *POGLUT1* cause Dowling-Degos Disease type 4; the hyperpigmentation associated with this autosomal dominant disease was previously attributed to altered Notch signaling, but our results suggest that this disease and other POGLUT1-associated phenotypes may be due to altered activity of CRUMBS proteins.

## Introduction

Glycosylation can regulate protein stability and function by ensuring efficient protein folding and by altering the binding affinity to interacting partners [1–7]. The biological importance of this type of protein modification is highlighted by dozens of human diseases caused by congenital disorders of glycosylation (CDGs), which are categorized based the chemical linkage, the added sugar, and the enzymes mutated in affected individuals [8–10]. Developmental defects in mice with mutations in glycosyltransferases have defined specific developmental roles for protein glycosylation in FGF and Notch signaling and in the composition of the extracellular matrix [11–18].

Epidermal Growth Factor (EGF) repeats are cysteine-containing motifs of about 40 amino acids found in many transmembrane and secreted proteins, including the Crumbs proteins and members of the Notch family receptors and ligands. EGF repeats can mediate ligand-receptor interactions and facilitate protein folding, and these interactions can be modified by glycosylation [4, 5]. EGF repeats can be modified by three types of O-linked glycosylation: O-fucosylation, O-GlcNAcylation and O-glucosylation. O-Fucose is added to the serine or threonine in the consensus C^2^-X-X-X-X-(S/T)-C^3^ (where C^*x*^ refers to the conserved cysteines in the EGF repeats) [19], O-GlcNAc to the serine or threonine in the putative consensus C^5^XXGX(S/T)GXXC^6^ [20] and O-glucose to the serine in the consensus sequence C^1^-X-S-X-(P/A)-C^2^ [21]. Specific enzymes add these sugars to EGF repeats: Protein O-fucosyltransferase 1 (POFUT1) [22], EGF-specific O-GlcNAc transferase (EOGT) [23] and Protein O-glucosyltransferase 1 (POGLUT1) [24], respectively. *POFUT1* and *POGLUT1* are essential for development in both *Drosophila* and mammals [13, 24–26].

POGLUT1 (also called KTELC1 or human CAP10-like protein 46KD (hCLP46) [27, 28]) is the only mammalian enzyme known to add O-glucose to the EGF repeats of NOTCH [24]. *Drosophila* Poglut1 (Rumi) was identified in a genetic screen based on its role in Notch signaling and was shown to modify the EGF repeats on the extracellular domain of Notch receptor. *Rumi* mutants have a temperature dependent defect in Notch signaling: at high temperature, the Notch receptor is not cleaved after binding to its ligand, preventing formation of the active Notch intracellular domain (NICD), whereas Notch proteolysis is normal at low temperature. O-glucosylation does not appear to affect ligand binding, and the data suggest it is required to couple binding of ligand to proteolytic activation of the receptor [24]. Mammalian POGLUT1 was identified based on homology to the *Drosophila* protein and was also shown to have O-glucosyltransferase activity [13, 29]. Inactivation of mouse *Poglut1* causes midgestation lethality with defects in neural tube development, somitogenesis, cardiogenesis, and vascular remodeling [13]. Knockdown of mouse *Poglut1* decreases Notch signaling in C2C12 cells, but does not strongly affect the levels of cell surface Notch protein or the binding of NOTCH to its ligand; instead, like the *Drosophila* protein, POGLUT1-dependent modification appears to affect mouse Notch activity at a step between ligand binding and S3 cleavage of NOTCH [13]. A number of other proteins contain predicted sites of POGLUT1 modification [13], including *Drosophila* Crumbs and mammalian CRUMBS1 and CRUMBS2.

Crumbs family proteins have essential, evolutionarily conserved roles in the organization and integrity of epithelia. Mammals have three members of the Crumbs family (CRUMBS1, CRUMBS2 and CRUMBS3). CRUMBS1 and CRUMBS2 have large extracellular domains that include multiple EGF-like repeats, and CRUMBS3 is a short membrane-anchored protein that includes the conserved cytoplasmic domain. Humans and mice that lack *Crumbs1* are viable but experience light-dependent retinal degeneration [30]. Mice lacking *Crumbs3* die shortly after birth due to defects in lung and intestinal epithelia [31]. Mouse *Crumbs2* mutants arrest at mid-gestation with a variety of morphological defects that have been attributed to a defective polarity in the epiblast [32].

We isolated an ENU-induced mutation called *wsnp* (*wing-shaped neural plate*) based on its striking abnormal morphology at midgestation, including a deficit of mesoderm and a completely open neural plate [33]. Here we show that *wsnp* is a null allele of *Poglut1* and that Notch signaling is almost completely blocked in *Poglut1* ^*wsnp*^ embryos *in vivo*. However, the phenotype of *Poglut1* ^*wsnp*^ and *Poglut1* ^-/-^ mutant embryos is more severe than the phenotype caused by complete loss of Notch signaling, suggesting that POGLUT1 has additional targets during mouse embryonic development. We noted that the early phenotype of *Poglut1* mutant embryos was similar to that described for *Crumbs2* [32] and strongly resembled that of another ENU-induced mutant characterized in the gene encoding Erythrocyte protein band 4.1l5 (Epb4.1l5), which can interact with the intracellular domain of Crumbs proteins [34–36]. Here we show that CRUMBS2 must be O-glucosylated by POGLUT1 for its activity during the mammalian gastrulation epithelial-to-mesenchymal transition (EMT). In the absence of POGLUT1, CRUMBS2 is trapped in the endoplasmic reticulum and is not trafficked to the apical plasma membrane. The data show that this trafficking defect causes a complete loss of CRUMBS2 function and argue that the loss of apical CRUMBS2 is responsible for the gastrulation defects seen in *Poglut1* null embryos.

## Results

### An ENU-induced null allele of *Poglut1* causes midgestation lethality with characteristic morphological defects

**Fig 1:**
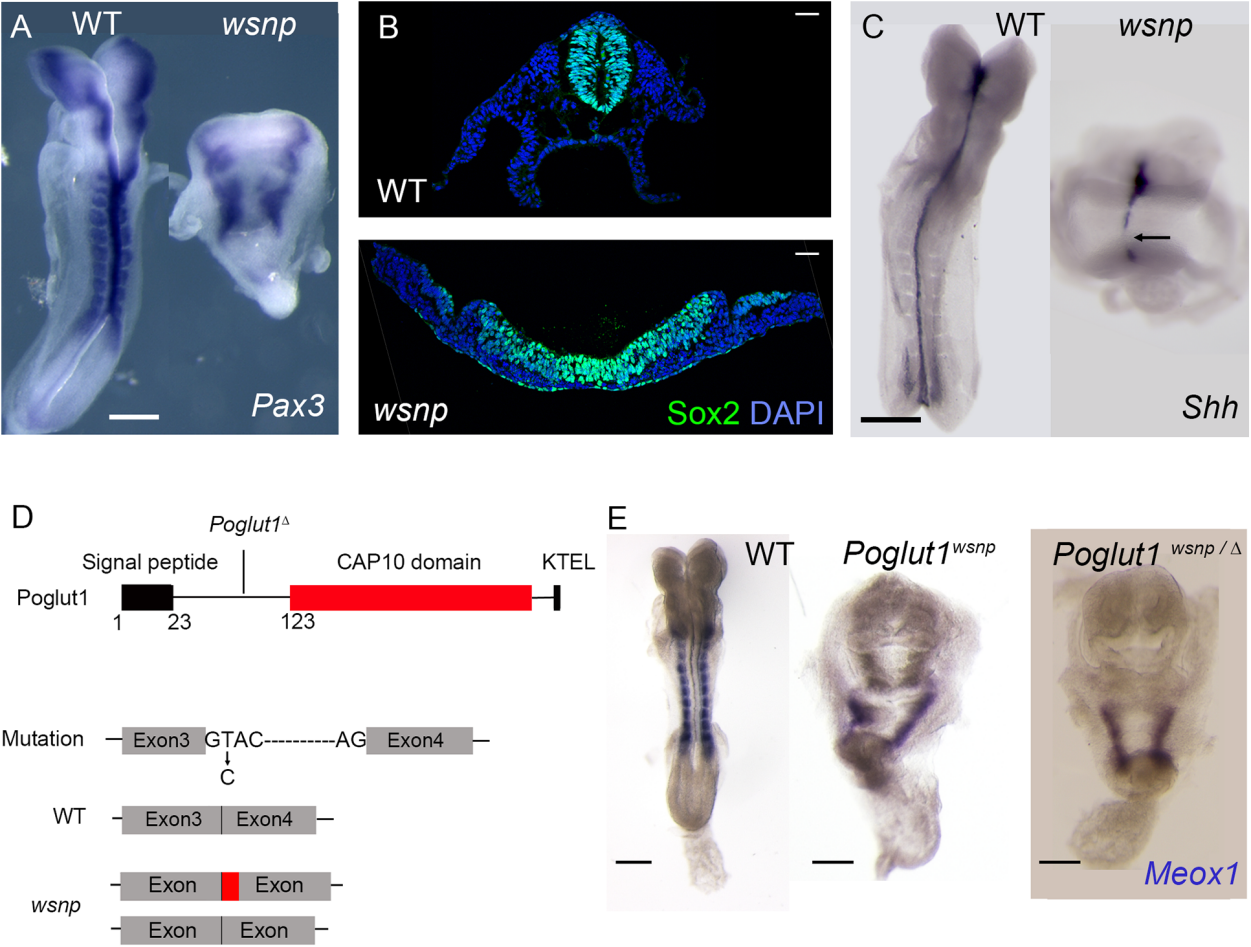
*wsnp* is a null allele of *Poglut1*. (A) *Pax3* is expressed in the dorsal neural tube and somites of WT E8.5 embryos. Neural tube closure fails in *wsnp* mutants, leading to the widely separated stripes of *Pax3* expression; no somite expression of *Pax3* is detected in the mutants. (B) Immunostaining for SOX2 expression in transverse sections of E8.5 neural epithelia. While the wild-type neural tube at trunk level is closed, the *wsnp* neural epithelium remains flat and fails to close. (C) *Sonic hedgehog* (*Shh*) expression at E8.5, showing a continuous midline in WT embryos and a discontinuous midline in *wsnp* mutants. (D) The Poglut1 protein includes a signal peptide, a CAP10 domain and an endoplasmic reticulum retention signal (KTEL). The *wsnp* allele harbors a splice site mutation that leads to multiple spliced products (either including a part of the intron 3 or skipping exon3), which both disrupt the enzymatic domain. Exon 4 is deleted in the *Poglut1* ^*Δ*^ allele leading to a frameshift and a premature stop codon. The line for the *Poglut1* ^*Δ*^ allele indicates where the wild-type protein sequence stops. (E) *Meox1* is expressed in the E8.5 paraxial mesoderm, which is segmented in WT, but not in *wsnp* homozygotes or in *Poglut1* ^Δ/wsnp^ transheterozygous embryos, demonstrating that *wsnp* fails to complement *Poglut1* ^*Δ*^. (A, C, E) Dorsal views, anterior up. Scale bars = 150 μm.

We isolated the *wing-shaped neural plate* (*wsnp*) mutant in a screen for ENU (N-ethyl N-nitrosourea)-induced recessive mutations that disrupt the morphology of the mid-gestation embryo [33, 37]. The mutants at E8.5 had a shortened anterior-posterior body axis, lacked Pax3+ somites (Fig 1A) and had a flat SOX2+ neural epithelium that failed to close and form a neural tube (Fig 1B). *Sonic hedgehog* (*Shh*) is expressed along the midline notochordal plate of wild-type E8.5 embryos, whereas *wsnp* mutants had discontinuous patches of midline *Shh* expression, demonstrating a disruption in specification or morphogenesis of the mesendoderm (Fig 1C). Despite the reduction in mesoderm-derived tissues in *wsnp* mutants, they formed a single primitive streak on the posterior side of the embryo, as seen by markers of Nodal and Wnt signaling (S1 Fig).

We mapped *wsnp* to a 407 kb interval between the D16Mit90 and D16Mit12 SSLP markers. Exonic sequences in this interval were captured and SOLiD sequencing identified a single nucleotide substitution in the interval (see Methods), a T to C transition in the splice donor site of intron 3 of *Poglut1*, an endoplasmic reticulum resident enzyme that adds O-glucose to EGF repeats of proteins [24]. The *wsnp* mutation resulted in abnormally spliced products that lacked most of the CAP10 catalytic domain and the KTEL ER-retention signal, and was therefore likely to be a strong loss-of-function allele (Fig 1D). We generated a null allele of the gene from embryonic stem cells from the International Mouse Knockout Project (*Poglut1* ^*Δ*^; Materials and Methods). *Poglut1* ^*wsnp/Δ*^ embryos showed the same midgestation lethality and abnormal morphology seen in *wsnp/wsnp* and *Poglut1* ^*Δ*^ /*Poglut1* ^*Δ*^ embryos, demonstrating that *wsnp* was a null allele of *Poglut1* (Fig 1E). The embryonic phenotype of the *wsnp* and *Poglut1* ^*Δ/Δ*^ homozygotes resembled that reported previously for a gene trap allele of the gene [13].

Because of its ubiquitous expression in the embryo (S2A Fig), we tested whether POGLUT1 activity was required in the epiblast-derived or extraembryonic tissues of the embryo. We generated embryos in which *Poglut1* was deleted specifically in the epiblast using the conditional allele derived from the International Mouse Knockout Project allele and the *Sox2-Cre* transgene [38]. *Poglut1 epiblast-deleted* embryos died before E9.0 and were indistinguishable from *wsnp* mutants, with reduced and unsegmented paraxial mesoderm and a flat neural plate (S2C Fig). Thus, POGLUT1 activity is required in the embryonic tissues for normal development.

### POGLUT1 is required for Notch signaling in the mouse *in vivo*

**Fig 2:**
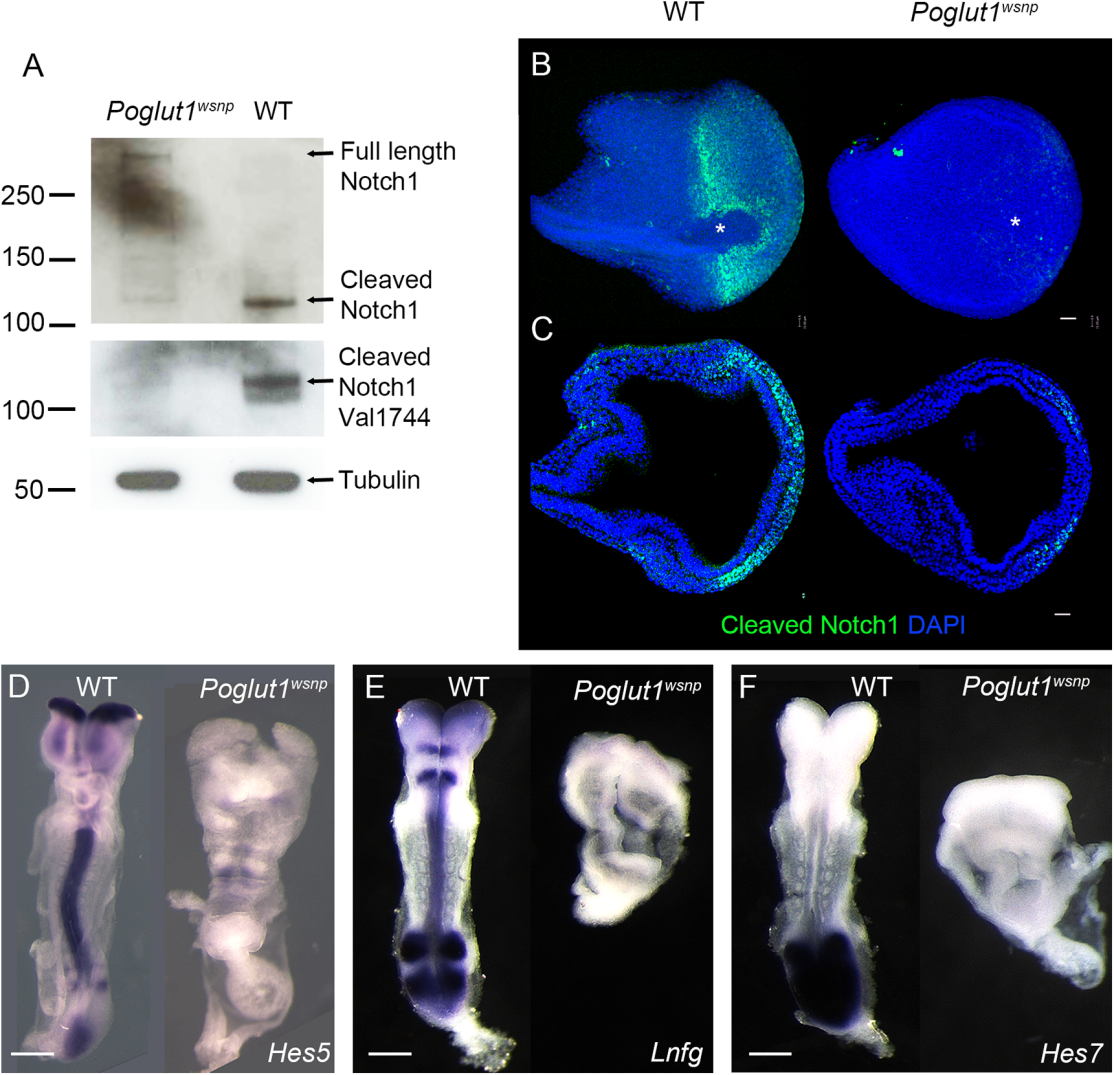
POGLUT1 is required for mammalian Notch signaling *in vivo*. (A) Western blots of wild type and *Poglut1* ^*wsnp*^ E8.5 embryonic lysates probed with antibodies for NOTCH1. Upper panel, an antibody that recognizes the intracellular domain of NOTCH1 shows the reduction of cleaved NOTCH1 in *Poglut1* ^*wsnp*^ mutants. In the lower panel, the Val1744 antibody that recognizes the active NOTCH1, shows the reduction of the active form in *Poglut1* ^*wsnp*^ mutants. α-TUBULIN is the loading control. (B) Whole mount immunostaining for active NOTCH1 at E7.75. View from the distal tip of the embryo (extended projection), anterior to the left, shows active NOTCH1 expression around the node (asterisk) in wild-type embryos, which is absent in *Poglut1* ^*wsnp*^ mutants. (C) Single optical transverse section through the primitive streak (to the right), showing active NOTCH1 expression in the nascent mesoderm in wild-type embryo and its reduction in *Poglut1* ^*wsnp*^ mutants. (D-F) Expression of the Notch pathway targets *Hes5* (D), *Lunatic Fringe* (E) and *Hes7* (F) in E8.5 wild-type and *Poglut1* ^*wsnp*^ embryos. (E, F) are dorsal views, anterior up. (D) is a ventral view with anterior up. Scale bars: 42 μm in B, C and 150 μm in D, E and F.

Knockdown of *Poglut1* in mammalian cell lines decreases Notch signaling [13] but a direct effect of POGLUT1 on Notch signaling has not been studied in an *in vivo* context. To determine whether Notch signaling was altered in *Poglut1* ^*wsnp*^ embryos, we tested whether NOTCH was correctly processed to form NICD, the active transcription factor form of the protein. In wild-type E8.5 embryos, most of the Notch protein was active, as assayed by the presence of active NOTCH1 in whole embryo lysates probed with the antibody that specifically recognizes the ϒ-secretase cleaved (S3 cleaved, Val1744) active NOTCH1 [39] (Fig 2A). The amount of NICD was greatly reduced (6.9 ± 1.3 fold) in *Poglut1* ^*wsnp*^ mutants (Fig 2A). In parallel, an antibody to the intracellular domain that detects both full-length and active NOTCH1 confirmed that active NOTCH1 was decreased in *Poglut1* ^*wsnp*^ mutants, while the full-length unprocessed form was more abundant in *Poglut1* ^*wsnp*^ embryos than in wild type (Fig 2A).

By immunostaining, NICD was detected around the node (Fig 2B) and in the nascent mesoderm cells emerging from the primitive streak in wild-type embryos (Fig 2C), similar to previous findings [40]. Active NOTCH1 was greatly reduced in *Poglut1* ^*wsnp*^ mutants by this assay, in either the distal view or in sections through the primitive streak (Fig 2B and 2C). Consistent with the lack of active NOTCH1 protein, the expression of direct transcriptional targets of the Notch pathway, *Hes5*, *Lunatic Fringe* and *Hes7*, was strongly reduced in *Poglut1* ^*wsnp*^ mutants compared to wild type at E8.5 (Fig 2D–2F).

### CRUMBS2 as a target of POGLUT1

Although the data show that POGLUT1 is required for normal Notch signaling in the mouse embryo, the phenotypes of *Poglut1* mutant embryos were more severe than the phenotypes of Notch pathway mutants. Mammals have four Notch proteins and multiple ligands, and compound mutants lacking all of the receptors have not been analyzed. However, mammals have only a single gene encoding the essential transcriptional NICD co-factor *Rbpjk* and two *Presenilin* genes that can cleave Notch proteins to their active form. *Rbpjk* mutants and *Presenilin* double mutants differentiate mesoderm-derived structures, including somites, and close their neural tubes [41, 42], unlike *Poglut1* ^*wsnp*^ embryos. Thus POGLUT1 appears to have additional, Notch-independent activities in the early mouse embryo.

**Fig 3:**
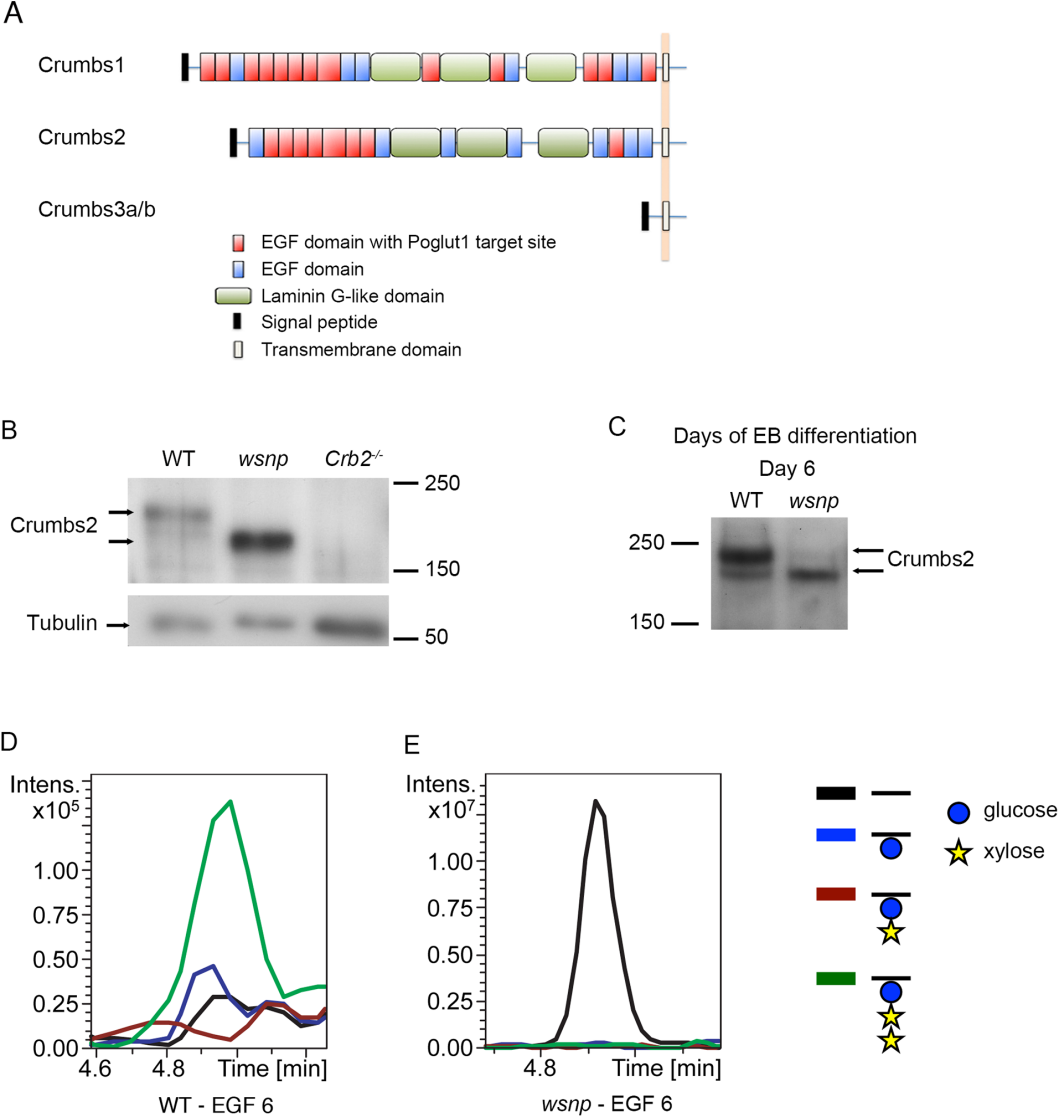
EGF repeat 6 in the purified full-length CRUMBS2 protein is O-glucosylated. (A) The domain organization of mammalian CRUMBS proteins. All three proteins have a short highly conserved cytoplasmic domain and a large variable extracellular domain. CRUMBS1 has 19 EGF repeats, 13 of which could be modified by POGLUT1, and CRUMBS2 has 15 EGF repeats, 8 of which could be modified by POGLUT1 based on its consensus recognition sequence [13, 48]. (B) Western blot of wild-type, *Poglut1* ^*wsnp*^ and *Crumbs2* ^*-/-*^ E8.5 embryos probed with a pan-CRUMBS antibody, showing the more rapid migration of CRUMBS2 in *Poglut1* ^*wsnp*^ embryos and the absence of this band in *Crumbs2* mutants. (C) Western blot for expression of endogenous CRUMBS2 in embryoid bodies at day 6 following differentiation from ES cells probed with pan-CRUMBS antibody. As in embryos, CRUMBS2 in *Poglut1* ^*wsnp*^ embryoid bodies migrated more rapidly than the protein in wild-type embryos. (D-E) Mass spectral analysis of O-glucosylation of full-length tagged CRUMBS2 purified from WT and *Poglut1* ^*wsnp*^ EBs differentiated for 2 days. Relative amounts of different glycoforms of the peptide ^258^SGERCEVDEDECASGPCQNGGQCL^281^ from EGF repeat 6 of CRUMBS2 were compared by generating Extracted Ion Chromatograms (EICs) from the mass spectral data. (D) The peptide from wild-type EBs was modified with O-glucose in the trisaccharide form (green line). The same peptide from *Poglut1* ^*wsnp*^ EBs (E) was unmodified by O-glucose (black line). Glucose is represented by a blue circle, xylose by a yellow star, and the peptide by a black line. Full mass spectra for the peptides from each sample are shown in S3B and S3C Fig.

In addition to the Notch family of receptors and ligands, over 40 proteins in the mouse genome contain predicted POGLUT1 modification sites [13]. Among these proteins, CRUMBS1 and CRUMBS2 have extracellular EGF repeats that could be modified by POGLUT1 (Fig 3A), and the *Crumbs2* ^*-/-*^ embryonic phenotype appeared to be similar to that of *Poglut1* mutants [32], making it a good candidate for a POGLUT1 target. Indeed, recent studies showed that *Drosophila* Crumbs can be modified by addition of O-glucose, although that modification is not required for the function of *Drosophila* Crumbs [43]. Consistent with a difference in post-translational modification, we found that CRUMBS2 protein from *Poglut1* ^*wsnp*^ mutant embryonic extracts at E8.5 migrated more rapidly than CRUMBS2 protein from wild-type embryos (Fig 3B).

### The extracellular domain of full-length CRUMBS2 is modified directly by POGLUT1 *in vivo*

To analyze whether CRUMBS2 was a direct target of POGLUT1 *in vivo*, we needed to purify microgram amounts of CRUMBS2 protein from wild type and mutants. We therefore generated wild-type and *Poglut1* ^*wsnp*^ mutant embryonic stem cells (ES cells) from mouse blastocysts under 2i+LIF conditions [44] and expressed full-length CRUMBS2 with a V5-tag in these cells. ES cells are not epithelial and therefore did not express high levels of endogenous CRUMBS2. After differentiation, wild-type embryoid bodies (EBs) at day 6, which is equivalent to E8.5 of mouse gestation, expressed endogenous CRUMBS2 (Fig 3C). EBs derived from *Poglut1* ^*wsnp*^ mutant ES cells also expressed endogenous CRUMBS2, but it migrated more rapidly than the protein derived from wild-type embryoid bodies (Fig 3C), as seen in embryos (Fig 3B). We therefore used wild-type and *Poglut1* ^*wsnp*^ EBs for biochemical analysis.

We purified the full-length V5-tagged CRUMBS2 from wild-type and mutant EBs (S3A Fig), and subjected the proteins to chymotrypic digestion and analyzed by nanoLC-MS/MS as previously described [45]. Analysis of a peptide derived from EGF repeat 6 of CRUMBS2 purified from wild-type embryoid bodies showed that it was modified by the addition of an O-glucose trisaccharide, in which the O-glucose was elongated by the addition of two xylose residues (Fig 3D; S3B Fig). This trisaccharide glycoform is also found on the EGF repeats of mammalian NOTCH1 containing the POGLUT1 consensus sequence for O-glucosylation [13, 21, 46]. This sugar modification was absent in EGF repeat 6 of CRUMBS2 purified from *Poglut1* ^*wsnp*^ embryoid bodies. Instead, the unmodified peptide was the only species detectable in the *Poglut1* ^*wsnp*^ mutants (Fig 3E; S3C Fig). These data demonstrate that EGF repeat 6 in the extracellular domain of the full-length CRUMBS2 protein was O-glucosylated by POGLUT1 *in vivo*.

### POGLUT1 is required for cell surface localization of CRUMBS2

**Fig 4:**
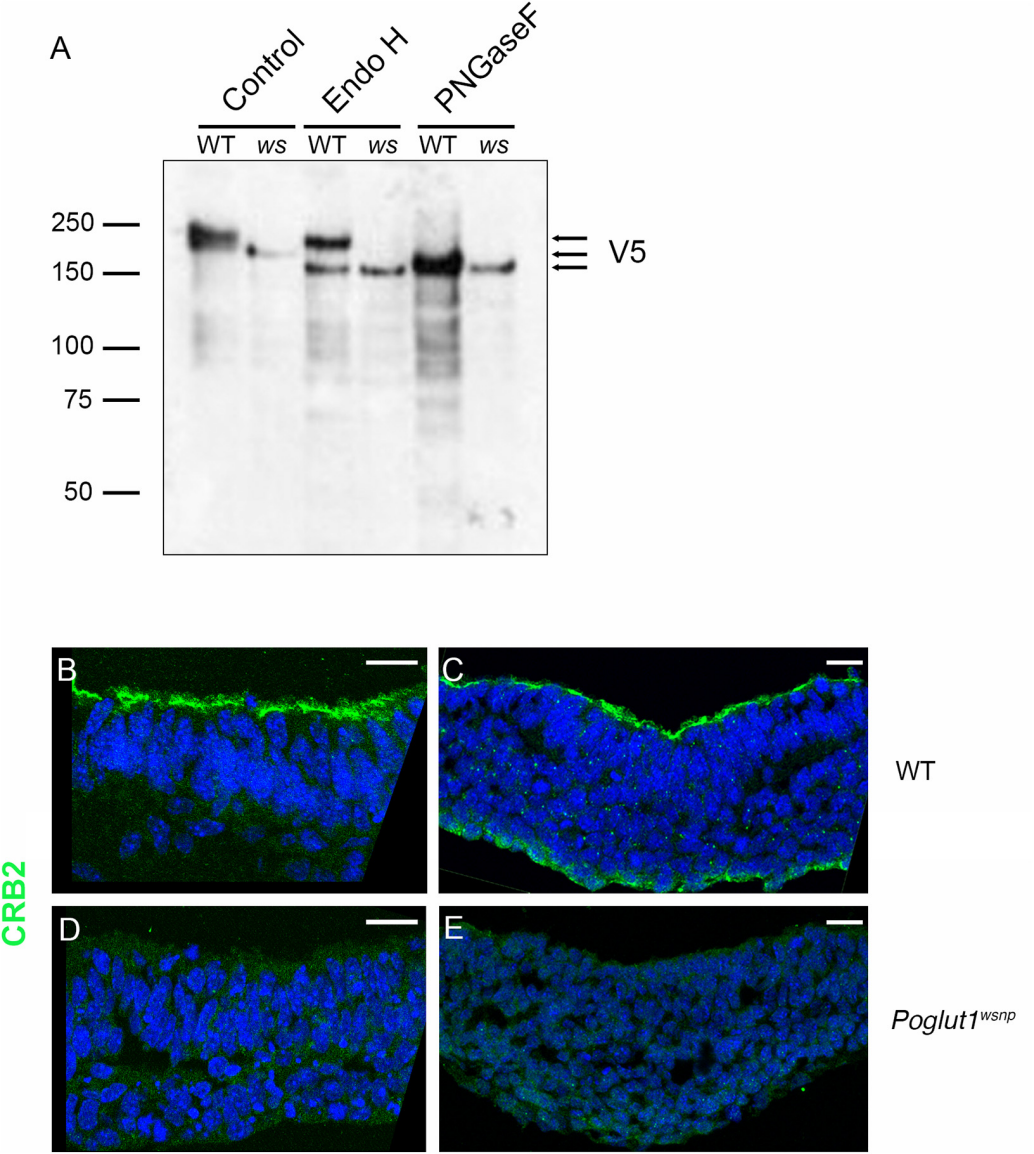
POGLUT1 is required for cell-surface localization of CRUMBS2. (A) Western blot of V5-tagged CRUMBS2 expressed in WT or *Poglut1* ^*wsnp*^ (*ws*) EBs after being subjected to PNGase F or Endo H digestions (Materials and Methods). EBs were differentiated for 5–6 days. Wild-type CRUMBS2 protein is modified with N-glycans sensitive to PNGase F, whereas CRUMBS2 from *Poglut1* ^*wsnp*^ EBs is modified with Endo H-sensitive N-glycans, consistent with trapping of the protein in the ER. (B-E) Immunolocalization of CRUMBS2 in transverse sections of E8.5 wild-type neural plate (B) and primitive streak (C). CRUMBS2 in transverse sections of the E8.5 *Poglut1* ^*wsnp*^ neural plate (D) primitive streak (E) is not localized to the apical cell surface (up). Scale bars: 20 μm.

The observed difference in migration on SDS-PAGE between CRUMBS2 protein from *Poglut1* ^*wsnp*^ and wild-type embryos and embryoid bodies (Fig 3B and 3C) was greater than could be accounted for simply by loss of O-glycosylation and suggested that there might be differences in N-glycosylation that takes place in the ER and the Golgi. To test this, we treated the CRUMBS2 protein purified from embryoid bodies with Endoglycosidase H (Endo H) or Peptide N-glycosidase F (PNGase F). Endo H removes high-mannose type N-glycans, which are found on immature proteins in the ER, while PNGase F removes all types of N-linked glycans, including those found on proteins that have moved through the Golgi to the cell surface. Treatment with PNGase F caused CRUMBS2 from both *Poglut1* ^*wsnp*^ and wild-type samples to shift to the same size (Fig 4A), confirming that the difference in migration is due to differences in N-glycosylation. Endo H treatment increased the mobility of the CRUMBS2 protein from *Poglut1* ^*wsnp*^ embryoid bodies, suggesting this protein was modified with high mannose type N-glycans and was likely localized to the ER (Fig 4A). In contrast, Endo H had no effect on the majority of CRUMBS2 from wild-type embryoid bodies, demonstrating that this protein had moved through the Golgi to the surface. A smaller portion of the wild-type protein was sensitive to Endo H, suggesting that a small pool of the wild-type protein was in the ER. These data demonstrate that mutant protein was modified with Endo H-sensitive N-glycans, while the majority of the wild-type protein was modified with complex-type N-glycans sensitive to only PNGase F. The results strongly suggest that the CRUMBS2 protein in *Poglut1* ^*wsnp*^ mutants is localized to the ER and that O-glucosylation of EGF repeats is required for CRUMBS2 transport to the Golgi, where subsequent processing to complex type N-glycan structures occurs.

By immunostaining, CRUMBS2 was detected in the apical plasma membrane of the E8.5 wild-type neural plate and primitive streak (Fig 4B and 4C). In contrast, there was no detectable apical CRUMBS2 in the *Poglut1* ^*wsnp*^ neural epithelium or primitive streak (Fig 4D and 4E), consistent with the biochemical finding that Crumbs2 was trapped in the ER when it was not O-glycosylated by POGLUT1.

CRUMBS1 was localized to the Golgi in both wild type and *Poglut1* ^*wsnp*^ mutants at this stage (S4A and S4B Fig), which suggested that trafficking of these two Crumbs proteins is differentially regulated in this tissue and that CRUMBS2 is the relevant target of POGLUT1 in the early mouse embryo.

### The gastrulation phenotypes of *Poglut1* ^*wsnp*^ and *Crumbs2* are indistinguishable

**Fig 5:**
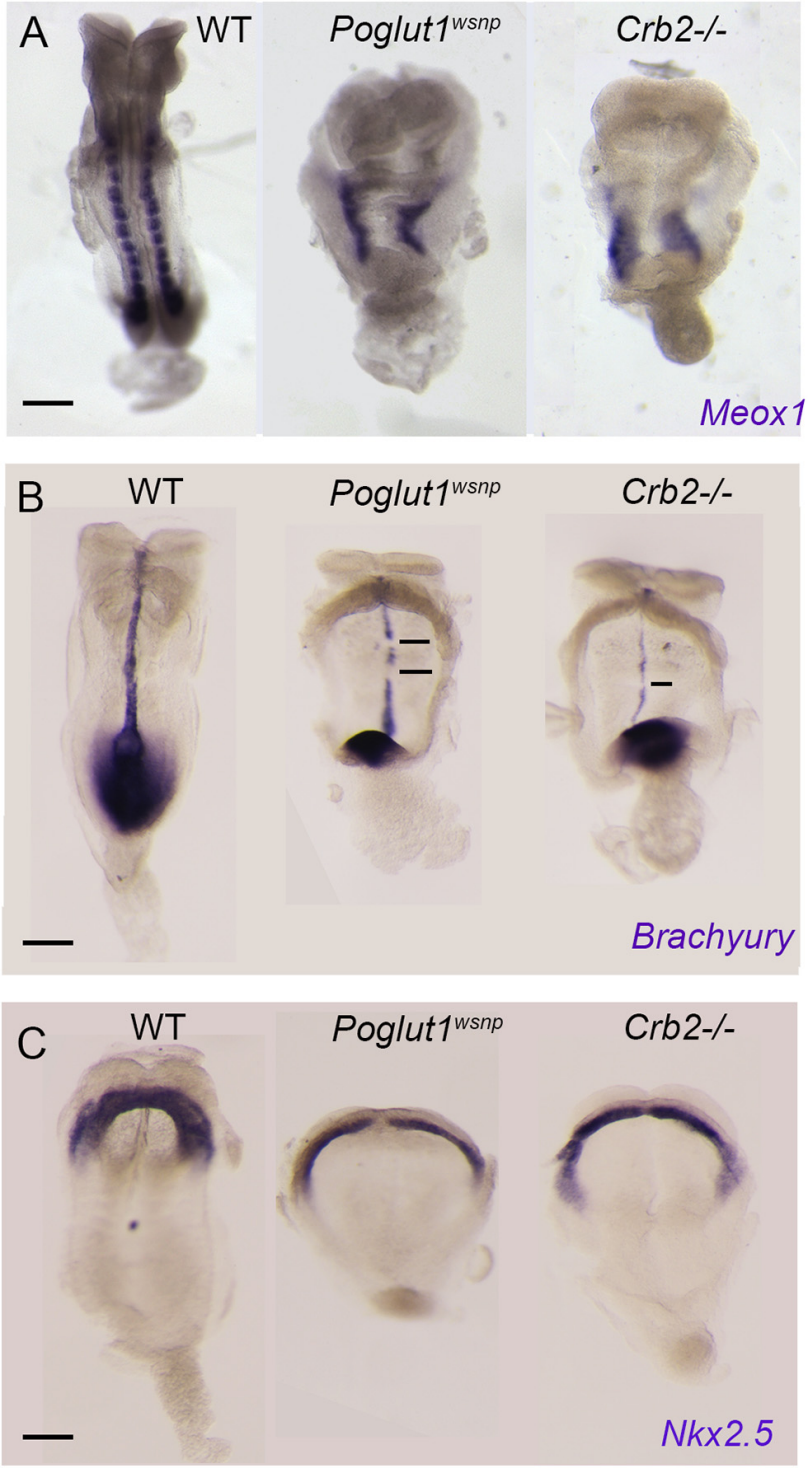
Indistinguishable phenotypes of early *Poglut1* ^*wsnp*^ and *Crumbs2* mutant embryos. (A) *Meox1* expression is reduced and unsegmented in the paraxial mesoderm of both mutants at E8.5 (dorsal views). (B) *Brachyury* expression at E8.5 shows a smaller primitive streak at the posterior end and a discontinuous midline in both mutants (ventral view, anterior up). (C) *Nkx2*.*5* expression shows the abnormal shape of the heart fields in the mutants at E8.5, ventral views. Anterior is up in all panels. Scale bars: 150 μm.

To test whether the loss of cell-surface CRUMBS2 could account for the phenotype of *Poglut1* mutant embryos, we compared the phenotypes of null alleles of the two genes using marker analyses. Like *Poglut1* mutants, E8.5 *Crumbs2* mutants have much less paraxial mesoderm, marked by expression of *Meox1*, than wild type (Fig 5A). Both *Poglut1* ^*wsnp*^ and *Crumbs2* mutants had a discontinuous midline, as marked by expression of *Brachyury* in axial mesoderm (Fig 5B). Although cardiac mesoderm was specified in *Crumbs2* and *Poglut1* mutants, as assayed by *Nkx2*.*5* expression, the E8.5 cardiac anlage was thinner and wider in the mutant than in wild type (Fig 5C) and the heart fields failed to fuse and form a single heart tube in both mutants. Despite these morphological similarities, the expression of a Notch target gene in the somites, *Uncx4*.*1*, appeared normal in the paraxial mesoderm of *Crumbs2* mutants (S5 Fig), whereas it is not expressed in *Poglut1* ^*wsnp*^ embryos, confirming that the Notch pathway is blocked by the absence of POGLUT1 but not the absence of CRUMBS2.

**Fig 6:**
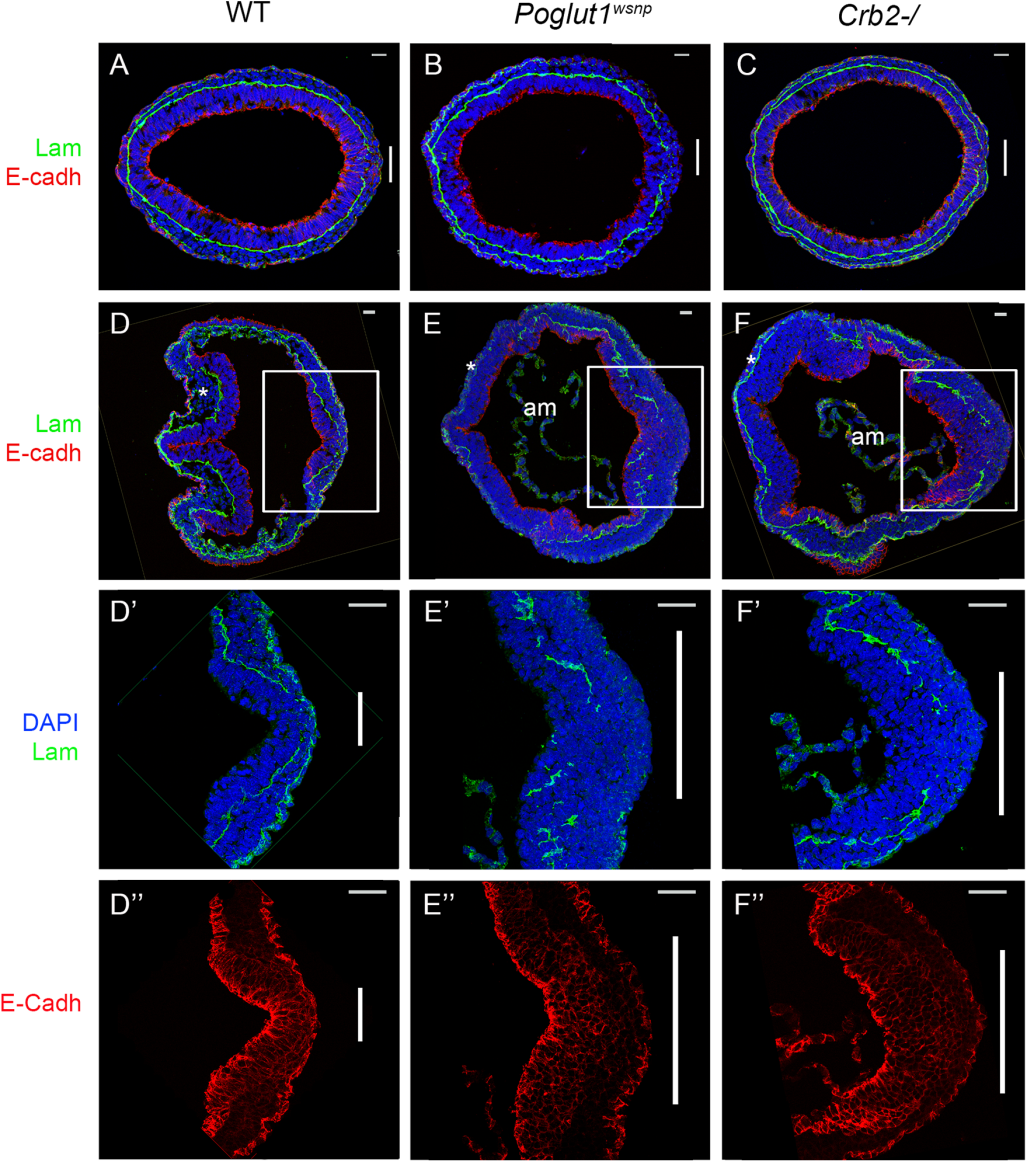
Indistinguishable gastrulation defects in *Poglut1* ^*wsnp*^ and *Crumbs2* ^*-/-*^ embryos. (A-C) Transverse sections through the primitive streak of early bud (E7.5) wild-type (A), *Poglut1* ^*wsnp*^ (B) and *Crumbs2* ^*-/-*^ (C) embryos immunostained for laminin (green) and E-Cadherin (red). The primitive streak (right) is marked by the break in the laminin-containing basement membrane (vertical bar). The mesodermal wings between the two laminin-containing basement membranes surround nearly the entire embryo in all three genotypes. (D-F) Transverse sections through E8.0 wild-type (D), *Poglut1* ^*wsnp*^ (E) and *Crumbs2* ^*-/-*^ (F) embryos at E8.0 (D, E, F). By E8.0 the mutants have less mesoderm (compare the head mesenchyme in WT and mutants, asterisks). am = amnion. D’, E’ and F’ and D”, E” and F” are higher magnification of the primitive streak (boxed in D, E, F) showing laminin expression (D’, E’ and F’) and E-Cadherin expression (D”, E” and F”). Both mutants have a broader streak as defined by the region of laminin breakdown. Both *Poglut1* ^*wsnp*^ and *Crumbs2* ^*-/-*^ mutants show an accumulation of E-cadherin+ cells in the vicinity of the primitive streak. Scale bars: A to F = 20 μm and D’ to F” = 40 μm.

The most prominent phenotype of both *Poglut1* ^*wsnp*^ and *Crumbs2* mutants was the shortened body axis accompanied by a deficit of mesoderm. Mesoderm cells arise during gastrulation in an epithelial-to-mesenchymal transition (EMT) at the primitive streak, in which cells delaminate from the epithelial epiblast layer at the site of breakdown of the basement membrane, down-regulate E-cadherin, acquire mesenchymal characteristics and begin to migrate around the circumference of the embryo. The primitive streak regions of *Poglut1* ^*wsnp*^ and *Crumbs2* mutants were comparable to wild-type at E7.5 (Fig 6A–6C). However, by E8.0 both the mutants had a broader streak than wild type, marked by the region of basement membrane breakdown at the streak (Fig 6D–6F). In both mutants, cells near the streak accumulated some ectopic laminin (Fig 6E’ and 6F’). In both *Poglut1* ^*wsnp*^ and *Crumbs2* ^*-/-*^ embryos, cells expressing E-cadherin accumulated at the primitive streak (Fig 6E” and 6F”), although some E-cadherin-negative cells were present in thin mesodermal wings (Fig 6E” and 6F”). The decreased number of mesoderm cells was apparent in laminin-stained transverse sections: in wild-type embryos, anterior head mesenchyme was present below the neural epithelium (Fig 6D) but there was very little anterior head mesenchyme present in both the mutants (asterisks in Fig 6E and 6F).

## Discussion

Our data confirm previous findings in cultured cells and show that POGLUT1 is required for mouse NOTCH1 activity *in vivo*, as it is in *Drosophila*. As in *Drosophila*, POGLUT1 is required for the cleavage of NOTCH1 and activity of the Notch signaling pathway *in vivo* in the midgestation mouse embryo. The effects of POGLUT1 on Notch are apparent early in development: activated NOTCH1 accumulates in cells of the nascent mesoderm immediately after they exit the primitive streak, presumably setting the stage for segmentation of the presomitic mesoderm [47]. In contrast, our data indicate that the earlier developmental phenotype of *Poglut1* mutants is caused by the loss of glucose modification of another EGF repeat-containing protein, the apical transmembrane protein CRUMBS2.

Notch extracellular domain is decorated with O-fucose, as well as O-glucose, and loss of mouse *Pofut1* phenocopies *Rbpjk* mutants. CRUMBS2 and CRUMBS1 also have putative POFUT1 consensus sites (11 and 9 sites, respectively) [11, 26, 48]. The stronger phenotype of mouse *Poglut1* than *Pofut1* mutants suggests that the two types of O-glycosylation have distinct effects on the activities of CRUMBS proteins.

Previous studies that defined the activities of enzymes that glycosylate EGF repeats used short fragments of proteins that contained a few EGF repeats expressed in cell lines [13, 21, 24]. In the experiments presented here, we showed that full-length CRUMBS2 protein is modified by addition of a glucose-xylose-xylose trisaccharide to EGF repeat 6 *in vivo*. Although we assayed only a single repeat, we predict that most or all of the 8 EGF repeats of CRUMBS2 that include the consensus sequence for modification are likely to be O-glucosylated.

In the absence of *O*-glucose modification, CRUMBS2 is trapped in the ER and fails to accumulate at the apical membrane of cells in the mouse embryo. This is different from the effect of *Poglut1* mutants on NOTCH: in both *Drosophila Rumi* mutants and mammalian *Poglut1* knockdown cell lines, O-glucosylation is not required for cell surface localization or ligand binding of NOTCH; instead, O-glucosylation is required for the proper conformation of NOTCH to allow efficient cleavage by metalloproteases (S2 cleavage) and presenilin (S3 cleavage) to generate the active NICD transcription factor [13, 24]. As POGLUT1 localizes to the endoplasmic reticulum, the lack of cell surface localization of CRUMBS2 in *Poglut1* mutants is likely to be due to a requirement for glycosylation of the EGF repeats of CRUMBS2 for its correct folding in the ER and subsequent trafficking to the Golgi and the cell surface. This hypothesis is supported by the accumulation of Endo H-sensitive forms of CRUMBS2 in *Poglut1* ^*wsnp*^ mutant embryoid bodies.

The EGF repeats in *Drosophila* Crumbs can also be modified by Rumi/POGLUT1 [43]. However, a mutant form of *Drosophila Crumbs* in which alanine replaced serine in all seven potential Rumi/POGLUT1 modification sites did not produce a mutant phenotype: homozygous S-to-A mutants were viable and appeared normal, and most of the mutant Crumbs protein localized normally to the membrane [43]. Thus glucose modification of CRUMBS2 is essential for its function in mammals but not in *Drosophila*. In *Drosophila*, *Rumi* mutants exhibit a Notch-dependent phenotype only when raised at higher temperatures [24, 43]. In contrast, POGLUT1-dependent modifications are essential for the activity of mammalian NOTCH. The more significant role of O-glucose addition in mammals may correlate with the more extensive POGLUT1-dependent modifications present on the mammalian proteins and may also reflect the higher body temperature of mammals. In all cases examined to date, O-glucose is extended to the trisaccharide form on all the EGF repeats of mammalian NOTCH [13, 21] and CRUMBS2 (this work). However, the predominant species of POGLUT1-modified EGF repeats in *Drosophila* Notch and Crumbs is glucose monosaccharide, which only occasionally is extended to the trisaccharide [24, 43].

POGLUT1-dependent modification of CRUMBS2 proteins is likely to be important in a variety of human diseases. Mutations in EGF repeats of human CRUMBS1, another likely substrate of POGLUT1, are found in retinitis pigmentosa and Leber congenital amaurosis [49–52], and could affect the glycosylation status of CRUMBS1 and its membrane localization in the eye. Mutations in human *CRUMBS2* are associated with some cases of congenital nephrosis [53, 54], suggesting that *POGLUT1* is also a candidate gene in this human syndrome. Whole exome sequencing has demonstrated that human *POGLUT1* mutations are responsible for a subset of the cases of Dowling-Degos Disease, an autosomal dominant hyperpigmentation disorder [55]. POGLUT1 is overexpressed in several human leukemia, breast cancer and endometrial cancer cell lines [28, 56–58]. It has been suggested that POGLUT1 mutations disrupt the activation of Notch signaling pathway in these diseases; our results suggest that POGLUT1-dependent Crumbs activity should also be considered.

## Materials and Methods

### Ethics statement

This work was approved by the Memorial Sloan Kettering Cancer Center IACUC (protocol number 02-06-013) and studies were conducted in accordance to their guidelines.

### Mouse strains

The *wsnp* allele was generated by ENU mutagenesis of C57/BL6J mice [33]. This allele harbors a T to C transition in the splice donor site of intron 3 of *Poglut1*, which creates an SfaNI restriction fragment length polymorphism. ES cells harboring the knock-out first allele were obtained from the International Mouse Knockout Project (HEPD0700_1_A09). They were injected into C57BL/6J blastocysts to generate chimeras, and chimeras were screened for transmission of gene trap allele (*Poglut1* ^*gt*^), which introduces the LacZ coding gene downstream of Exon 3. The conditional allele (*Poglut1* ^*flox*^) was generated by crossing *Poglut1* ^*gt*^ to actin-Flip mice [59]. The null allele (*Poglut1* ^*Δ*^) was generated by crossing conditional allele to *CAG-Cre* [60]. The conditional allele of *Crumbs2* [61] was crossed to *CAG-Cre* (Jax) to generate the null allele (*Crumbs2* ^*-/-*^). *Crumbs1* ^*rd8*^ [62] and *Sox2-Cre* [38] have been described. Mice carrying the *nodal-lacZ* knock-in allele [63] was a gift from Elizabeth Robertson and the TOPGAL mice [64] were provided by Elaine Fuchs.

### Mapping and identification of *wsnp*

The *wsnp* mutation was mapped to a 407 kb interval between D16Mit90 and D16Mit12 simple sequence length polymorphism (SSLP) markers by meiotic recombination. Genomic DNA was purified from three mutants pooled together, and exonic DNA across the genomic interval was enriched using Agilent SureSelect solution based technology with custom target capture. Samples were multiplexed and bar-coded for SOLiD sequencing. Sequencing reads were aligned to the C57BL/6 reference genome using SHRiMP [65].

### Phenotypic analysis

Whole mount in situ hybridization and LacZ staining was performed as published [36]. For in situ hybridization embryos were dissected in ice-cold PBS-0.4%BSA and fixed in 4% paraformaldehyde overnight. Following a series of dehydration and rehydration in methanol series, embryos were hybridized with the corresponding in situ probes, washed and developed using BM-purple. For β-galactosidase activity, embryos were dissected in ice cold PBS and fixed in freshly prepared fixative solution (5mM EGTA, 0.2% glutaraldehyde and 2mM MgCl_2_ in 0.1M phosphate buffer (pH 7.3)) for 10–15 minutes. Following washes in detergent rinse (2mM MgCl_2_, 0.02% Nonidet P-40 and 0.01% sodium deoxycholate in 0.1M phosphate buffer), the embryos were stained with X-GAL (1mg/ml X-GAL, 5 mM potassium ferricyanide) in detergent rinse) overnight. Following staining, the embryos were rinsed in PBS and fixed in 4%PFA for an hour before imaging.

### Immunostaining

Embryos were dissected in ice cold PBS-BSA and fixed in 4% PFA for one hour at room temperature for immunostaining or over-night at 4°C for in-situ hybridization. The embryos were embedded in OCT (optimal cutting temperature) and cryosectioned at 10–12 μm thickness. Immunostaining on frozen sections was performed as published [36]. Primary antibodies were diluted in blocking buffer and incubated overnight at 4°C. The secondary antibodies were diluted in blocking buffer and incubated for 1 hour at room temperature. DAPI was included in the secondary incubation.

For whole mount active NOTCH1 immunostaining, embryos were dissected in ice cold PBS-BSA and fixed overnight in 4% PFA/PBS at 4°C, dehydrated in methanol and stored at -20°C overnight. Following rehydration, antigen unmasking was performed in Vector unmasking solution (H-3300 Vector labs) at 98°C for 10 minutes. After reaching room temperature embryos were washed in MilliQ water and incubated in acetone at -20°C for 8 minutes. Next the embryos were washed and incubated in blocking buffer overnight at 4°C (Blocking buffer = 10% goat serum, 5% BSA, 0.3% Triton-X100 in PBS). The embryos were incubated in anti-cleaved NOTCH1 antibody (1:1000) for 2 days. Following 4–5 washes with blocking buffer, the embryos were incubated in secondary antibody overnight at 4°C. The embryos were washed extensively before mounting in glass-bottom dishes for confocal imaging.

The following antibodies were used: anti-CRUMBS1 and anti-CRUMBS2 were obtained from Jane McGlade (Hospital for Sick Children, Toronto) and were used at 1:100 and 1:50 respectively [66]; Anti-pan-CRUMBS was a kind gift from Ben Margolis (University of Michigan, Ann Arbor) and was used at 1:200 [67]. Commercially available antibodies used were: anti-SOX2 (Santa Cruz 1:100), anti-E-CADHERIN (Sigma 1:200), anti-LAMININ (Sigma 1:200) and anti-active NOTCH1 (Val 1744—Cell Signaling 1:1000). Confocal microscopy was performed using a Leica-Inverted SP5 or Leica-Upright SP5 laser, point-scanning confocal microscope. Confocal datasets were analyzed using the Volocity software package (Improvision).

### Western blot analysis

Embryos were dissected in ice cold PBS and snap frozen on dry ice. The embryos were lysed in RIPA buffer with protease inhibitor added. The lysates were equilibrated on ice for 20 minutes, after which they were sonicated (3 X 30 seconds). Lysates were incubated for 10 minutes in ice and then centrifuged to remove the cell debris. The supernatant was mixed with 2X SDS loading dye (1:1) and loaded on an SDS-PAGE for analysis. The dilutions for primary antibody were: anti-pan-CRUMBS (Ben Margolis [64], 1:2000), anti-Cleaved-NOTCH1 (Val 1744—Cell Signaling 1:1000), anti-NOTCH1 (Abcam 1:1000) and anti-V5 (Invitrogen 1:5000).

### Cloning of tagged CRUMBS2

The *Crumbs2* cDNA was synthesized from wild-type embryos harvested at E8.5. Full-length *Crumbs2* was cloned into the Gateway vector pDEST40 to generate *Crumbs2* with His and V5 tags at its C-terminal. This sequence was sub-cloned with the tags into the EcoR1 site of the pCAGGS vector to generate pCAGGS-*Crumbs2* for high expression in embryonic stem cells.

### Generation of ES cell lines expressing full-length tagged CRUMBS2

ES cells were derived using the 2i protocol from both wild type and *Poglut1* ^*wsnp*^ blastocysts [44], were eventually weaned off iMEFs and were cultured in KnockOut DMEM (Gibco) supplemented with 15% fetal bovine serum (HyClone), 2 mM L-glutamine (Gibco), 0.1 mM β-mercaptoethanol (Gibco), 0.1 mM non-essential amino acids (Gibco), 1 mM sodium pyruvate (Gibco), 1% v/v penicillin and streptomycin (Gibco), 1,000 units LIF (Millipore), 1 μM PD0325901 and 3 μM CHIR99021 (Stemgent). To generate stable cells lines expressing tagged full-length CRUMBS2, wild-type and *Poglut1* ^*wsnp*^ ES cells were electroporated with pCAGGS-*Crumbs2* DNA linearized by cutting with ScaI and a circular *PGK-Puro-pA* plasmid [68] that confers a transient puromycin resistance. Stable lines were selected with puromycin (Invitrogen) using published protocols [69]. Fifteen independent colonies were screened for expression of tagged full length CRUMBS2 both by immunofluorescence and western blots analysis with the anti-V5 antibody. One cell line each for wild type and *Poglut1* ^*wsnp*^ was selected based on high expression levels and was used for protein purification.

### Embryoid body (EB) differentiation

ES cells were trypsinized and re-suspended at 10^6^ ES cells per 10 ml media in non-adherent conditions (10 cm bacterial petri dishes (VWR) coated with Sigmacote). EBs were cultured in the absence of LIF and 2i in DMEM medium containing 15% fetal bovine serum, 2 mM L-glutamine (Gibco), 0.1 mM β-mercaptoethanol (Gibco), 0.1 mM non-essential amino acids (Gibco), 1 mM sodium pyruvate (Gibco), 1% v/v penicillin and streptomycin (Gibco). The medium was replaced every day. To determine the time course of endogenous CRUMBS2 expression, EBs were harvested daily from day 1 to day 6.

### Purification of CRUMBS2 protein

ES cells were cultured in large (6X15 cm) plates and differentiated to embryoid bodies. Wild-type and *Poglut1* ^*wsnp*^ mutant embryoid bodies were harvested 2 days after induction of differentiation from ES cells and snap frozen. These were re-suspended in RIPA buffer with 2% DDM (n-Dodecyl β-D-maltoside) and Roche protease inhibitor and subjected to three cycles of freeze-thaw to increase the release of membrane protein, then sonicated for 4 times for 30 seconds each and incubated at 4°C for one hour with continuous shaking. The lysates were clarified by centrifugation for 20 minutes at 15,520 g. The supernatant was incubated with anti-V5 antibody (1:1000) for 4 hours at 4°C shaking. This mixture was incubated with magnetic nickel beads (Invitrogen) for 2 hours at 4°C shaking. The beads were rinsed four times in RIPA lysis buffer, and the beads were heated to 95°C in 2XSDS loading buffer to elute the protein. The eluted protein was run on a 7% SDS-PAGE. The gel was washed 3 times in HPLC purified water and then stained with Pierce gel code blue stain reagent for 5–10 minutes. Following staining, the bands were cut out, subjected to chymotrypsin digest and used for mass spectrometric analysis.

### Cleavage of N-glycans by PNGase F and Endo H

After 5 days of differentiation, wild-type and *Poglut1* ^*wsnp*^ EBs were lysed in sample buffer containing SDS and β-mercaptoethanol, and denatured by boiling for 5 min. After cooling, NP-40 was added and samples were diluted in PBS to final concentrations of 1% NP-40, <1%SDS and 1% β-mercaptoethanol. Samples were then incubated with 5U of Peptide-N-Glycosidase F (PNGase F), prepared as described [70] or 25mU of Endoglycosidase H (Endo H) (Roche) overnight at 37°C. Western blotting was performed to detect the removal of N-glycans from V5-tagged CRUMBS2 protein.

### Mass spectrometric analysis

Bands containing purified mouse CRUMBS2-V5 were reduced, alkylated, and subjected to in-gel chymotryptic digest as described previously [45]. Peptides were analyzed by nanoLC-MS/MS as previously described [21]. Glycosylation of peptides was identified by using neutral loss searches, and Extracted Ion Chromatograms (EICs) were generated to compare relative amounts of each glycoform of the relevant peptides.

## Supporting Information

S1 Fig Nodal and Wnt signaling at the streak are normal in *Poglut1* ^*wsnp*^ mutants. Both *Nodal-lacZ* expression (A) and Wnt reporter activity (TOPGAL) (B) are on the posterior side of E7.5 *Poglut1* ^*wsnp*^ embryos, as in wild type, although the length of the streak is shorter along the proximal-distal axis than in wild type. Lateral views, anterior to the left, distal down. Scale bars: 150 μm.
(TIF)

S2 Fig *Poglut1* is broadly expressed in the early embryo and is required in the epiblast. (A) Ubiquitous expression of *Poglut1* in the epiblast-derived tissues from E6.5 to E8.5, visualized by β-galactosidase activity in *Poglut1* ^*gt/+*^ embryos; lateral views, anterior left. (B) Trans-heterozygous *Poglut1* ^*wsnp/gt*^ embryos arrest at approximately E8.5 with the same morphology seen in *wsnp* homozygotes. (C) *Meox1* expression in wild type and *Poglut1 epiblast-deleted* embryos at E8.5. *Poglut1 epiblast-deleted* embryos have less paraxial mesoderm than wild type and are indistinguishable from *Poglut1* ^*wsnp*^ and *Poglut1* ^-/-^ embryos. (B, C) Dorsal views; anterior up. Scale bars: 150 μm.
(TIF)

S3 Fig EGF repeat 6 of mouse CRUMBS2 is *O*-glucosylated in wild-type EBs but not in *Poglut1* ^*wsnp*^ EBs. (A) Western blot of V5-tagged full-length CRUMBS2 extracted from WT and *Poglut1* ^*wsnp*^ ES cells differentiated to EBs, probed with anti-V5 antibody, showing the more rapid migration of tagged CRUMBS2 in *Poglut1* ^*wsnp*^ embryoid bodies compared to the protein from wild-type EBs. (B) Chymotryptic peptides from full-length CRUMBS2 purified from WT EBs were analyzed by mass spectrometry as described in Methods. The top panel shows a full MS spectrum of ions eluting at 4.9 minutes. The ion labeled [M+3H]^3+^ (m/z 1048.0) matches the mass for the triply charged form of the peptide ^258^SGERCEVDEDECASGPCQNGGQCL^281^ from EGF repeat 6 modified with a glucose trisaccharide. CID fragmentation of the ion generates the MS2 spectra in the bottom panel, which reveals sequential neutral losses of the two xyloses and glucose from the glycopeptide. Several fragment ions (b- or y-ions) of peptide are shown confirming its identity. (C) Chymotryptic peptides from full-length Crumbs2 purified from *Poglut1* ^*wsnp*^ EBs were analyzed as in B. The top panel shows a full MS spectrum of ions eluting at 5.0 minutes. The ion labeled [M+3H]^3+^ (m/z 905.9) matches the mass for the triply charged form of the same peptide from EGF repeat 6 with no modification. CID fragmentation of the ion generates the MS2 spectra in the bottom panel. Several fragment ions (b- or y-ions) of the peptide are shown that confirm the identity of the peptide. Glucose is represented by a blue circle and xylose is represented as a yellow star, and the peptide by a black line. Red diamonds in MS spectra indicate ions chosen for CID fragmentation, and blue diamonds indicate the location of the parent ion fragmented in the MS2 spectra. The blue underlined S in the peptide sequence indicates the serine residue of the consensus sequence for O-glucosylation.
(TIF)

S4 Fig CRUMBS1 is localized to the Golgi in the early embryo. (A) Immunostaining for CRUMBS1 (green) in transverse sections through the primitive streak of wild-type and *Poglut1* ^*wsnp*^ embryos at E8.5. (B) The same image as in (A), including the red channel that shows the localization of GM130. CRUMBS1 is localized to the Golgi, as judged by its colocalization with GM130. CRUMBS1 localization is not altered in *Poglut1* ^*wsnp*^ mutants at this stage. Scale bars: 40 μm.
(TIF)

S5 Fig NOTCH1 is active in *Crumbs2* mutants. (A) Western blots for cleaved NOTCH1 in lysates from E8.5 wild-type, *Poglut1* ^*wsnp*^ and *Crumbs2* embryos. Cleaved NOTCH1 was reduced in *Poglut1* ^*wsnp*^ mutants, but unaltered in *Crumbs2* mutants. (B) *Uncx4*.*1* at E8.5 is expressed in the caudal half of each somite in wild type, but is undetectable in *Poglut1* ^*wsnp*^ mutants (n = 3). In contrast, *Uncx4*.*1* is expressed in a striped pattern in *Crumbs2* mutants (n = 3). Dorsal view, anterior up. Scale bar: 150 μm.
(TIF)
