# Supplementary figures and images for: Protein O-Glucosyltransferase 1 (POGLUT1) Promotes Mouse Gastrulation through Modification of the Apical Polarity Protein CRUMBS2
Source: PLoS Genet. 2015 Oct 23;11(10):e1005551. doi: 10.1371/journal.pgen.1005551 (PMC4619674; doi:10.1371/journal.pgen.1005551)

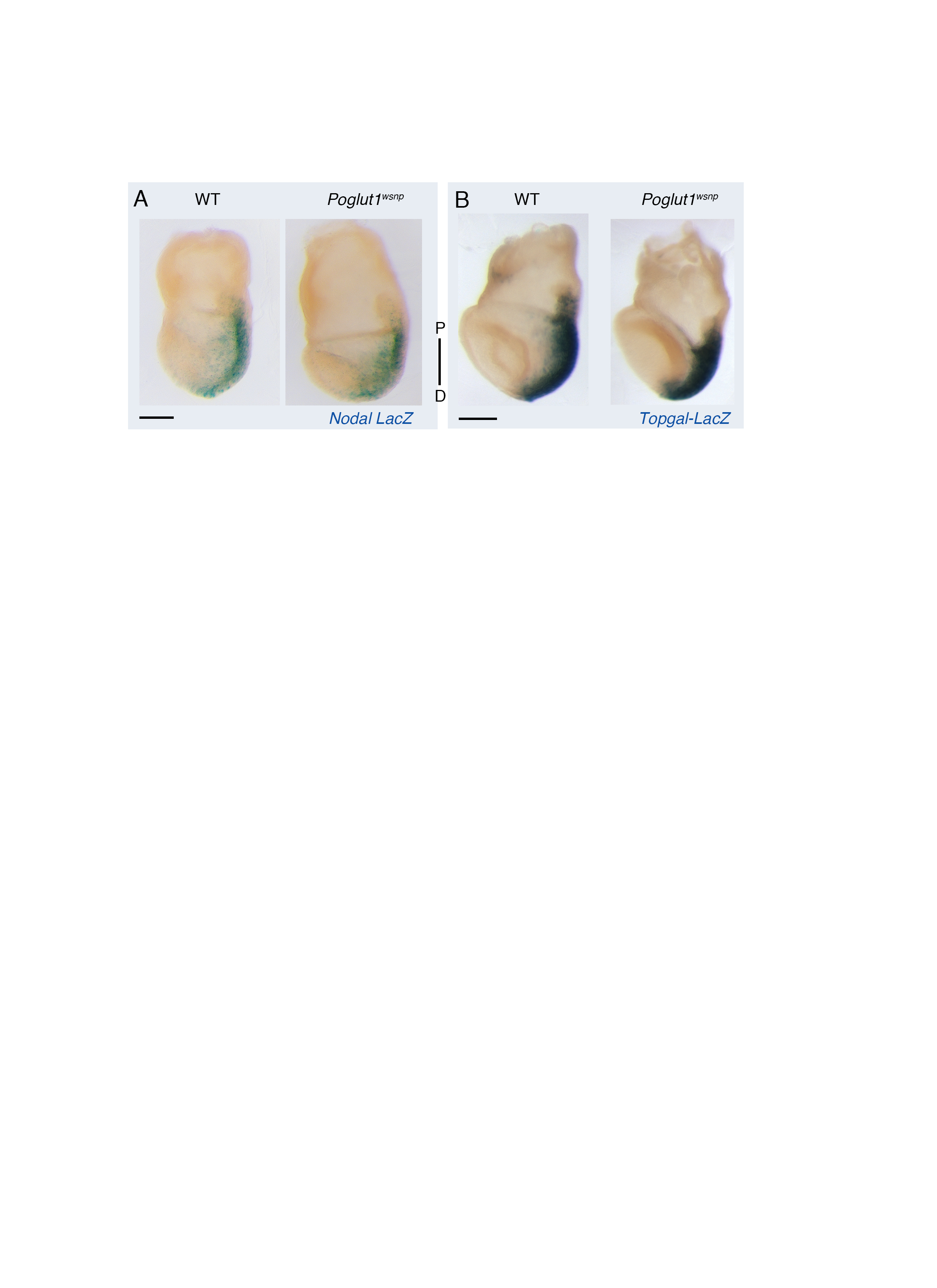

Supplement: S1 Fig — Both Nodal-lacZ expression (A) and Wnt reporter activity (TOPGAL) (B) are on the posterior side of E7.5 Poglut1 wsnp embryos, as in wild type, although the length of the streak is shorter along the proximal-distal axis than in wild type. Lateral views, anterior to the left, distal down. Scale bars: 150 μm. (TIF) [file pgen.1005551.s001.tif]

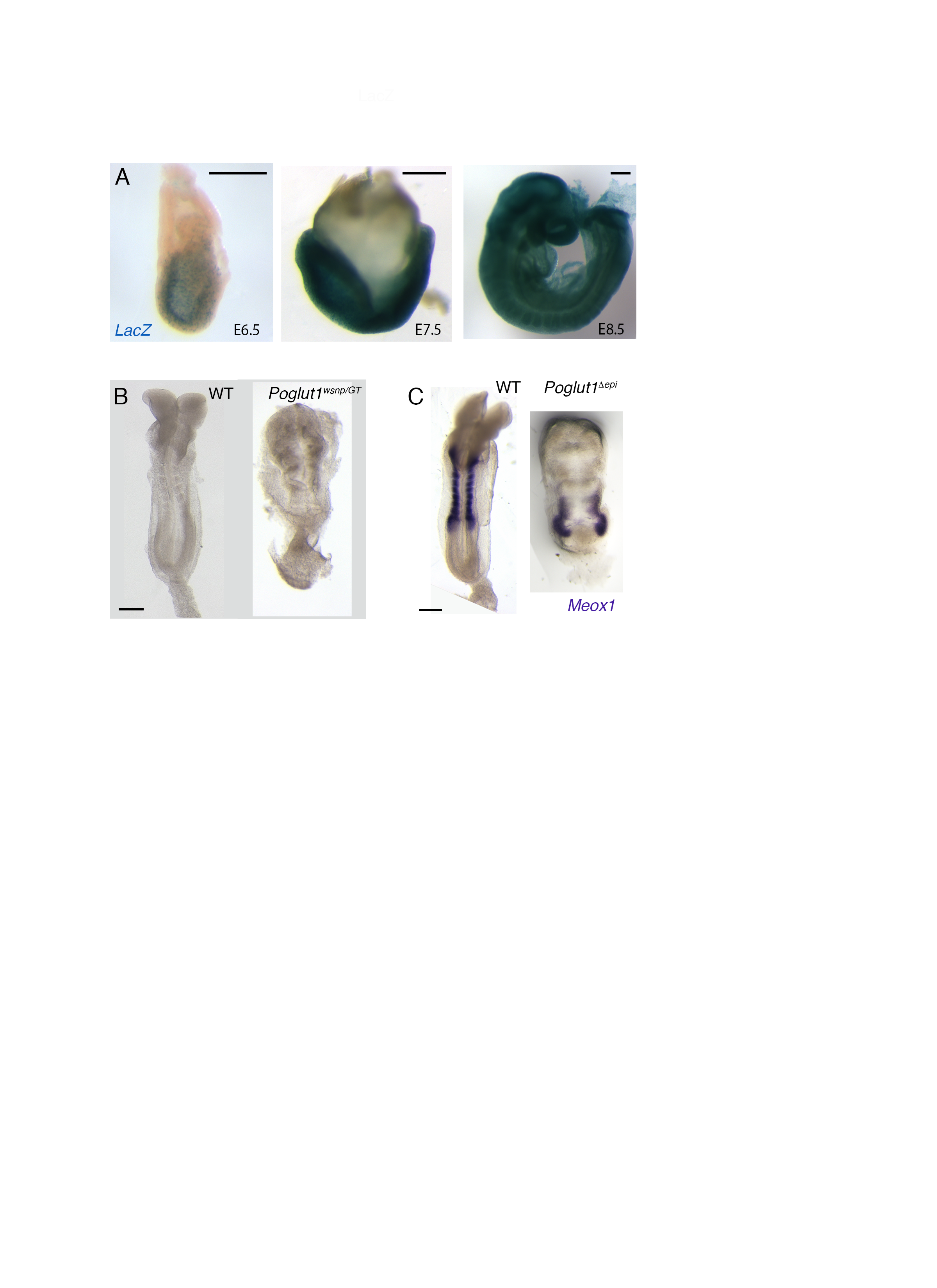

Supplement: S2 Fig — (A) Ubiquitous expression of Poglut1 in the epiblast-derived tissues from E6.5 to E8.5, visualized by β-galactosidase activity in Poglut1 gt/+ embryos; lateral views, anterior left. (B) Trans-heterozygous Poglut1 wsnp/gt embryos arrest at approximately E8.5 with the same morphology seen in wsnp homozygotes. (C) Meox1 expression in wild type and Poglut1 epiblast-deleted embryos at E8.5. Poglut1 epiblast-deleted embryos have less paraxial mesoderm than wild type and are indistinguishable from Poglut1 wsnp and Poglut1 -/- embryos. (B, C) Dorsal views; anterior up. Scale bars: 150 μm. (TIF) [file pgen.1005551.s002.tif]

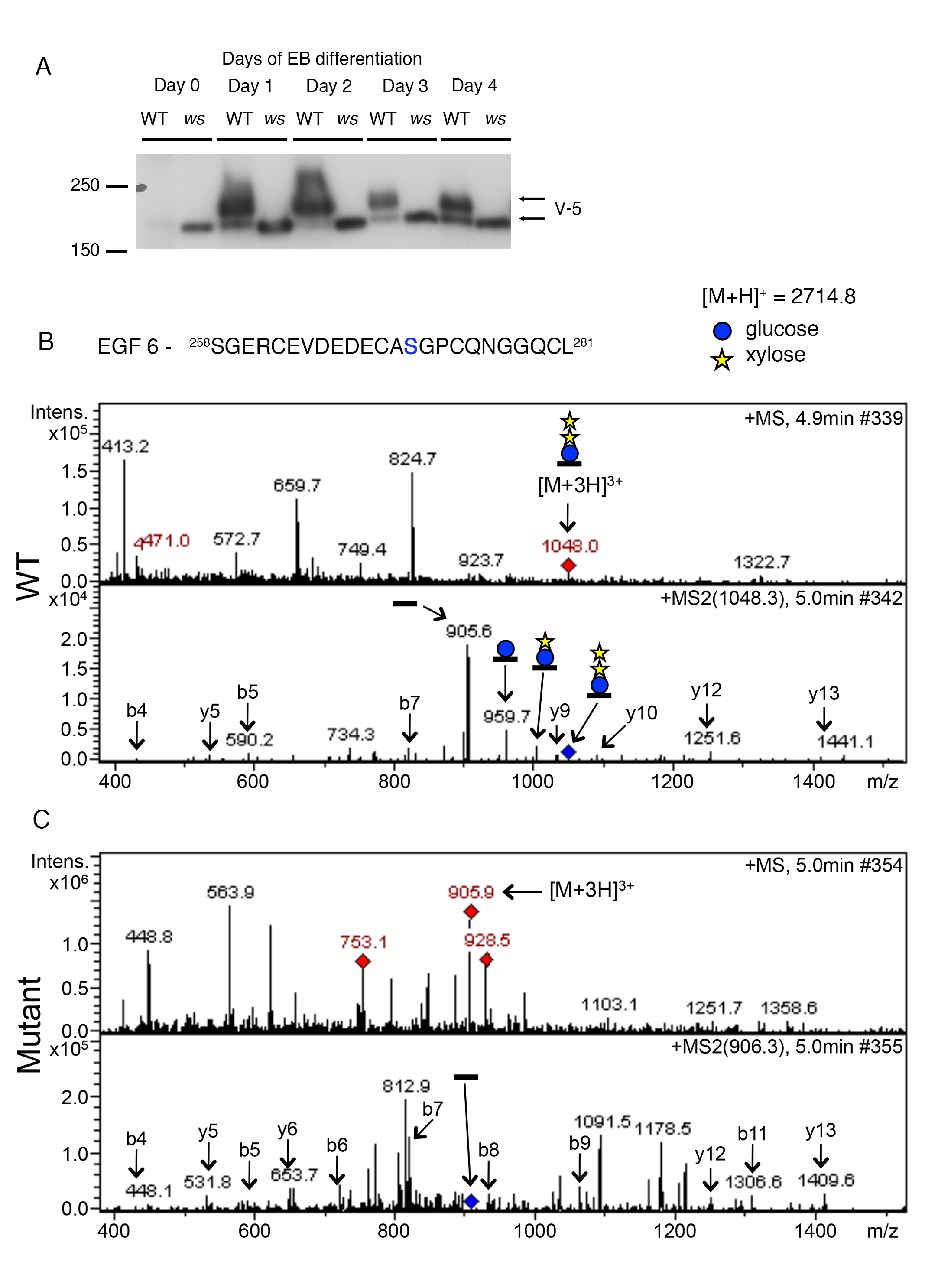

Supplement: S3 Fig — (A) Western blot of V5-tagged full-length CRUMBS2 extracted from WT and Poglut1 wsnp ES cells differentiated to EBs, probed with anti-V5 antibody, showing the more rapid migration of tagged CRUMBS2 in Poglut1 wsnp embryoid bodies compared to the protein from wild-type EBs. (B) Chymotryptic peptides from full-length CRUMBS2 purified from WT EBs were analyzed by mass spectrometry as described in Methods. The top panel shows a full MS spectrum of ions eluting at 4.9 minutes. The ion labeled [M+3H]3+ (m/z 1048.0) matches the mass for the triply charged form of the peptide 258SGERCEVDEDECASGPCQNGGQCL281 from EGF repeat 6 modified with a glucose trisaccharide. CID fragmentation of the ion generates the MS2 spectra in the bottom panel, which reveals sequential neutral losses of the two xyloses and glucose from the glycopeptide. Several fragment ions (b- or y-ions) of peptide are shown confirming its identity. (C) Chymotryptic peptides from full-length Crumbs2 purified from Poglut1 wsnp EBs were analyzed as in B. The top panel shows a full MS spectrum of ions eluting at 5.0 minutes. The ion labeled [M+3H]3+ (m/z 905.9) matches the mass for the triply charged form of the same peptide from EGF repeat 6 with no modification. CID fragmentation of the ion generates the MS2 spectra in the bottom panel. Several fragment ions (b- or y-ions) of the peptide are shown that confirm the identity of the peptide. Glucose is represented by a blue circle and xylose is represented as a yellow star, and the peptide by a black line. Red diamonds in MS spectra indicate ions chosen for CID fragmentation, and blue diamonds indicate the location of the parent ion fragmented in the MS2 spectra. The blue underlined S in the peptide sequence indicates the serine residue of the consensus sequence for O-glucosylation. (TIF) [file pgen.1005551.s003.tif]

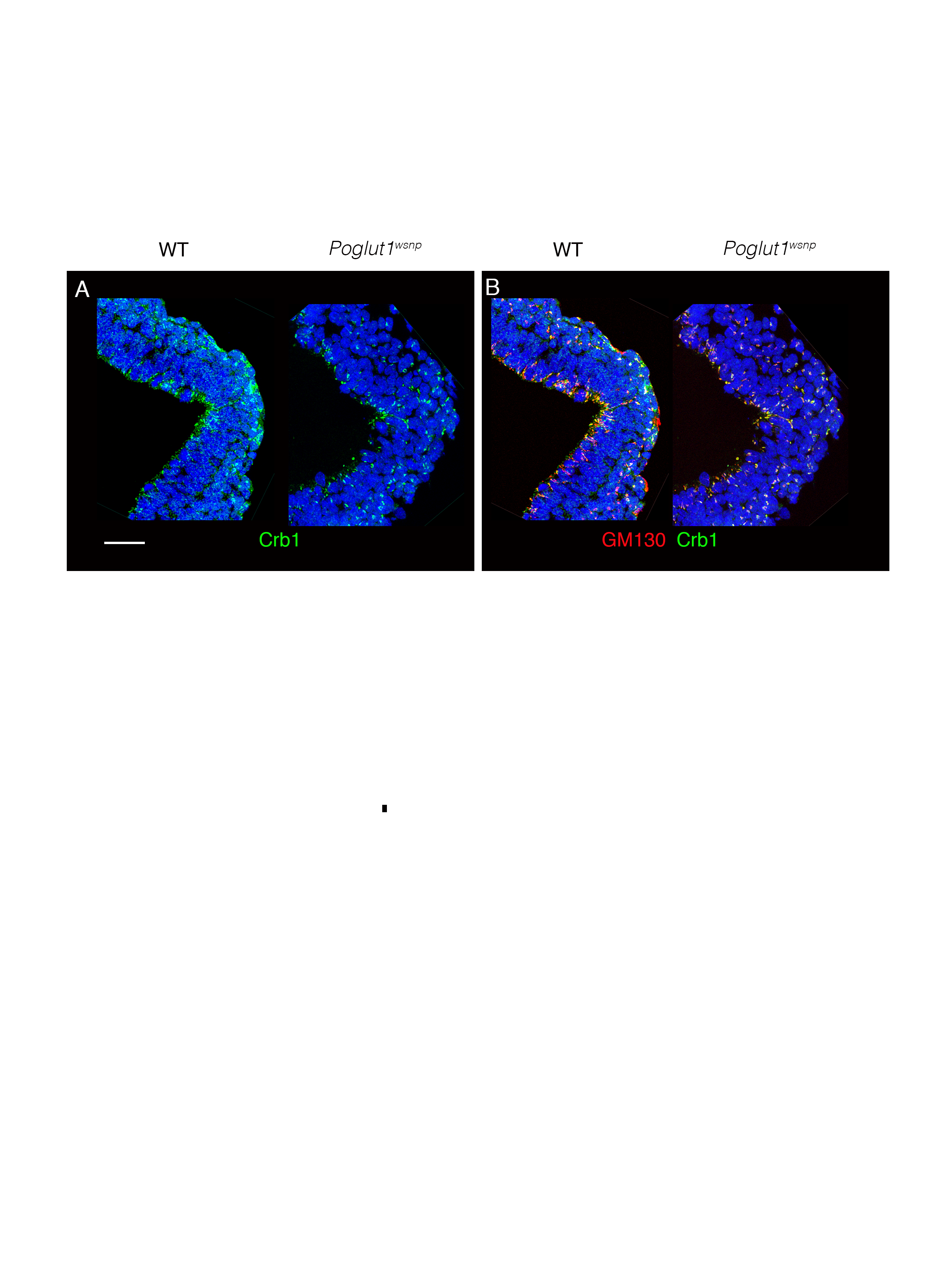

Supplement: S4 Fig — (A) Immunostaining for CRUMBS1 (green) in transverse sections through the primitive streak of wild-type and Poglut1 wsnp embryos at E8.5. (B) The same image as in (A), including the red channel that shows the localization of GM130. CRUMBS1 is localized to the Golgi, as judged by its colocalization with GM130. CRUMBS1 localization is not altered in Poglut1 wsnp mutants at this stage. Scale bars: 40 μm. (TIF) [file pgen.1005551.s004.tif]

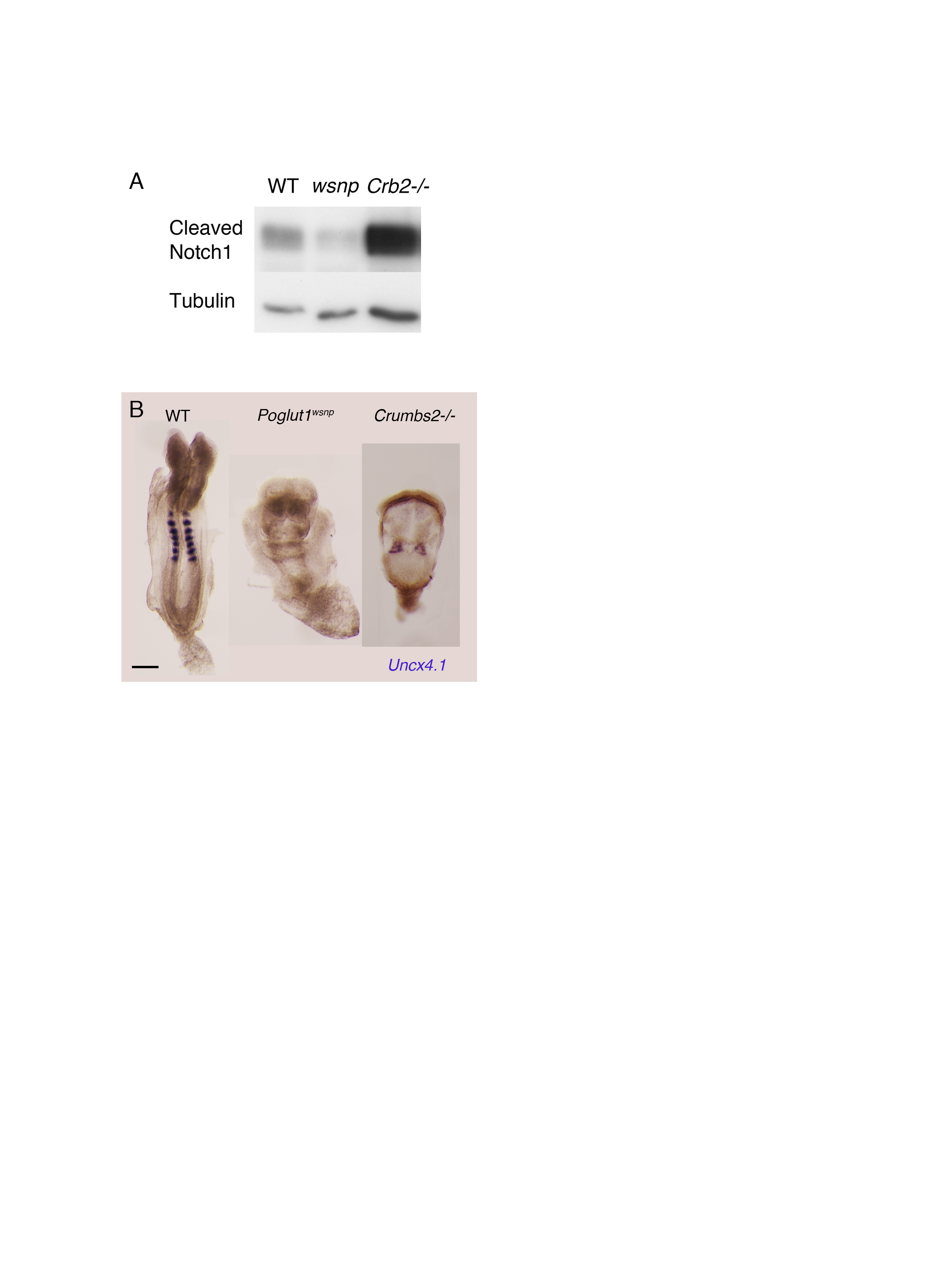

Supplement: S5 Fig — (A) Western blots for cleaved NOTCH1 in lysates from E8.5 wild-type, Poglut1 wsnp and Crumbs2 embryos. Cleaved NOTCH1 was reduced in Poglut1 wsnp mutants, but unaltered in Crumbs2 mutants. (B) Uncx4.1 at E8.5 is expressed in the caudal half of each somite in wild type, but is undetectable in Poglut1 wsnp mutants (n = 3). In contrast, Uncx4.1 is expressed in a striped pattern in Crumbs2 mutants (n = 3). Dorsal view, anterior up. Scale bar: 150 μm. (TIF) [file pgen.1005551.s005.tif]
